# A myofibre model for the study of uterine excitation-contraction dynamics

**DOI:** 10.1038/s41598-020-72562-x

**Published:** 2020-10-01

**Authors:** Uri Goldsztejn, Arye Nehorai

**Affiliations:** 1 Department of Biomedical Engineering, Washington University in St. Louis, St. Louis, 63130 USA; 2 Department of Electrical and Systems Engineering, Washington University in St. Louis, St. Louis, 63130 USA

**Keywords:** Computational models, Biomedical engineering, Electrical and electronic engineering

## Abstract

As the uterus remodels in preparation for delivery, the excitability and contractility of the uterine smooth muscle layer, the myometrium, increase drastically. But when remodelling proceeds abnormally it can contribute to preterm birth, slow progress of labour, and failure to initiate labour. Remodelling increases intercellular coupling and cellular excitability, which are the main targets of pharmaceutical treatments for uterine contraction disorders. However, the way in which electrical propagation and force development depend on intercellular coupling and cellular excitability is not fully understood. Using a computational myofibre model we study the dependency of electrical propagation and force development on intercellular coupling and cellular excitability. This model reveals that intercellular coupling determines the conduction velocity. Moreover, our model shows that intercellular coupling alone does not regulate force development. Further, cellular excitability controls whether conduction across the cells is blocked. Lastly, our model describes how cellular excitability regulates force development. Our results bridge cellular factors, targeted by drugs to regulate uterine contractions, and tissue level electromechanical properties, which are responsible for delivery. They are a step forward towards understanding uterine excitation-contraction dynamics and developing safer and more efficient pharmaceutical treatments for uterine contraction disorders.

## Introduction

Every year, there are about 15 million preterm births and 26 million induced labours worldwide, and these statistics are rising^1–4^. Preterm births, defined as births before 37 weeks of gestation, are associated with morbidity and mortality of the newborn, and they impose a large financial and emotional burden on the family^5,6^. Labour contractions are induced in response to delayed initiation of labour or inefficient labour progression, and in cases of post-partum hemorrhage^7^. However, labour induction increases the risks of complications and the need for interventions during birth. Complications during labour induction are one of the most common reasons for medical litigation^8^.

Labour results from myometrial excitation and contraction. In labour, action potential (AP) trains originate at alternating sites near the fundus and the placenta, and then propagate towards the cervix. These traveling APs form depolarization waves that progressively increase the intracellular calcium concentrations in the uterine smooth muscle cells (USMC). The intracellular calcium buildup produces contractions of increasing strength that raise the intrauterine pressure until the fetus is delivered^9^.

Tocolytic agents can arrest uterine contractions and delay preterm births, and uterotonic agents enhance uterine contractions and induce birth, but both have limited efficacy and frequent serious side effects. Safer and more efficient therapeutics could be developed if we had a better understanding of the electromechanical uterine activity^10^.

Uterine electrophysiology has been studied through multiple approaches. Experimental studies in-vitro and in-vivo have identified and characterized many of the electrophysiological components of USMCs that support electrical conduction and mechanical contraction in the myometrium. Moreover, many of the pathways that mediate the effects of tocolytic and uterotonic drugs have been identified^11^. However, experimental restrictions, coupled with the large amount of resources required for electrophysiological experimentation, have limited the exploratory possibilities.

Computational models of uterine electrophysiology have recently emerged and offer a complementary research approach. Existing models simulate either detailed ionic currents and contraction mechanisms in isolated cells or simplified depolarization and contraction waves at the whole tissue level. The cellular level models^12–14^ are useful in studying AP morphologies and calcium dynamics. However, these models exclude intercellular dynamics and therefore cannot be used to study electrical conduction and consequent mechanical activity.

Whole organ models, on the other hand, are useful for studying geometrical trends of excitation and contraction, and for solving the forward and inverse problems of uterine electrophysiology^15–18^. These models, however, require drastic reductions of the cellular models and can miss dynamically regulated cellular responses.

In this study, we developed a myofibre model to study the regulatory mechanisms of electrical conduction, conduction block, and force development. This study characterizes the propagation of the electromechanical wave in the myometrium. Our results introduce a theoretical framework that is useful to interpret experimental observations. Moreover, our model bridges between existing cellular level models and whole organ models. This bridge is necessary to understand the interplay between the cellular targets of drugs developed to regulate uterine contractions, and the tissue and organ level electromechanical properties responsible for delivery.

Our model consists of a series of cells, described by a detailed ionic current model, coupled longitudinally. The intercellular interactions are described by a system of equations that governs the electromechanical wave propagation.

We simulated the propagation of an electrical stimulus along the myofibre to study the effects of intercellular coupling and cellular excitability on uterine electrophysiology and force development. We made four observations: (1) Intercellular coupling determines the conduction velocity (CV) and remodels the AP. (2) Force development has a binary dependence on intercellular coupling: the force developed is constant under high intercellular coupling, and only a passive tension is developed under low intercellular coupling. (3) Increased cellular excitability can recover conduction that was blocked by low intercellular coupling, but it cannot recover the reduction in CV. Lastly, (4) Force development has a discontinuous dependency on cellular excitability.

Our results contribute a step forward in understanding the responses to tocolytic and uterotonic drugs. The mechanisms we explored, remodel in preparation for parturition. These mechanisms, in turn, are targeted by all the available uterotonic and tocolytic drugs. Consequently, better understanding of these mechanisms is necessary to develop more efficient and safer drugs.

## Results

### Model overview

**Figure 1:**
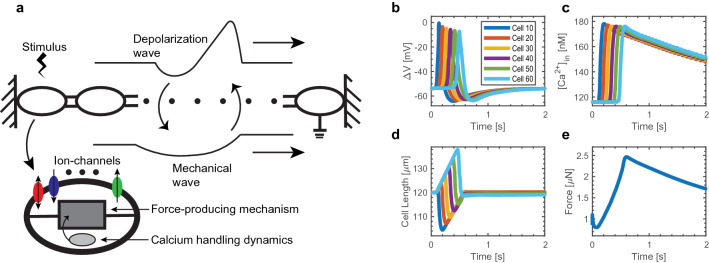
Myofibre model overview and validation. (**a**) A graphic representation of the model. The myofibre model (upper) consists of 70 serially connected cells (bottom). The detailed ionic current model, calcium handling dynamics, and force-producing mechanism are schematically represented. The upper and lower waveforms are schematic representations of the traveling depolarization and contraction waves, respectively. (**b**) The action potentials (AP) obtained at various cells along the myofibre. Downstream cells activate later in time. The AP amplitude decays slightly as it travels along the first cells and then stabilizes. (**c**) The intracellular calcium traces for the same cells as in (**a**). These traces present rapid upstrokes and slow decay rates. (**d**) The cellular length over time for the same cells as in (**a**). Cells with higher concentrations of myosin in force-producing states contract, while their relaxed neighbours dilate to satisfy the isometric condition. (**e**) The force developed by the myofibre in response to the depolarizing stimulus. The cellular response to a depolarization stimulus is long lasting and decays monotonically until it returns to its base value after 12 s (Supplementary Fig. S1).

We model a myofibre as a longitudinal concatenation of cells. Our multiscale model combines a detailed USMC model with a novel electromechanical myofibre model (Fig. 1a). Each cell in the myofibre includes a detailed ionic current model, calcium handling dynamics, actin-myosin interactions, and a force-producing mechanism. The myofibre model consists of 70 cells serially connected. We chose to model 70 cells in the myofibre since this length is sufficient to contain the entire depolarization and contraction waveforms, yet not too long to make the simulations computationally expensive. To exclude end effects, the first and last 10 cells of the myofibre are not considered for analysis.

The intercellular interactions are modeled using a system of differential equations, the electrical propagation is modeled through the cable equation, while the mechanical transduction is modeled by a system of 3*n* equations, where *n* is the number of cells in the myofibre. The electrical activity modulates the mechanical activity through the calcium dynamics, actin-myosin cycling, and cellular contraction model. Conversely, the mechanical activity modulates the electrical activity by varying the geometrical parameters in the cable equation. In particular, the mechanical wave arises from contracting and relaxing cells through the myofibre, which have varying lengths depending on their contractile state. This effect is taken into account through the spatial component of the wave equation. The complete formulation of our model appears in the “Methods” section.

We stimulate the myofibre from one end with a 30 ms long depolarization stimulus of − 5 pA/pF. This stimulus produces a single AP that propagates along the myofibre. Although APs in the contracting myometrium usually appear in bursts, the propagation of a single AP is useful to study the mechanisms responsible for the excitability and contractility of the myometrium. Considering a single AP simplifies the analysis by excluding the effects related to the properties of the burst, such as the firing rate. Additionally, many parameters used to quantify excitability and contractility, such as conduction velocity and action potential duration, are not well defined for a burst of APs.

Our model recapitulates experimental observations. The single cell model is based on the models by Tong et al.^12^ and Testrow et al.^13^, as well as on the previous models by Yang et al.^19^ and Hai and Murphy^20^. The developed myofibre model is based on Newton’s laws and recapitulates physiological behaviours. The AP waveforms obtained have physiological morphologies^21^, and propagate with experimentally observed conduction velocities^22^ (Fig. 1b). Additionally, the intracellular calcium transients (Fig. 1c), cellular contraction dynamics (Fig. 1d), and contractile force development (Fig. 1e) behave as expected, based on previously reported experimental recordings^12,20,21,23,24^.

### CV and AP morphology dependence on intercellular coupling

Intercellular coupling is achieved through gap junctions (GJ), which form channels that connect the cytosol of two adjacent cells and allow the flow of ions and small molecules. GJs consist of two connexons, each formed by six connexins, anchored to each of the connected cell’s membranes. Connexin 26, 32, 37, and 43 form myometrial GJs^25^. The expression level of GJs, as well as the type of connexins used to form those GJs, regulate the intercellular resistivity (IR)^26^.

The healthy myometrium remodels to induce parturition^25,27^. During pregnancy, connexins are expressed at a basal level, providing minimal intercellular coupling and preventing electrical conduction. Before birth, connexin expression and GJ formation is upregulated. Intercellular coupling increases, allowing APs to propagate across cells. This process, however, may go amiss, contributing to either premature contractions or slowly progressing labours^28^.

We simulated electrical conduction along a myofibre under different intercellular coupling conditions. In our model, intercellular coupling is determined by the IR between adjacent cells, and intercellular coupling is inversely related to IR. We varied the IR between the cells in the myofibre (as detailed in the “Methods” section) and determined its effects on conduction velocity, cellular depolarization, and conduction block.

Conduction velocity is defined as the propagation velocity of the depolarizing wave through the myometrium. This velocity has been reported to usually range between 1 and 3 cm/s in-vivo, but it has also been reported to be as high as 12 cm/s^22^. Cellular depolarization is driven mostly by a rapid calcium influx and is characterized by its maximal upstroke slope $\left( {\max}_{t} \frac{\partial v_{\mathrm{m}}}{\partial t}\right)$ and the duration of its upstroke. These two parameters define the ability of a USMC to propagate an AP. In our simulations, the time difference between the depolarization of adjacent cells is much shorter than the upstroke duration, so the upstroke section of the AP drives the propagation. The duration of the upstroke is measured from when the cell achieves 5% of the maximal depolarization until it achieves its maximal transmembrane voltage. Conduction block is defined as the inability of the stimulus to traverse the entirety of the myofibre.

**Figure 2:**
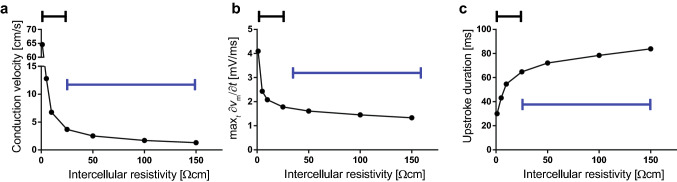
Electrical conduction characteristics with respect to intercellular resistivity (IR). (**a**) Conduction velocity (CV) with respect to IR. CV decreases with increasing IR until block occurs at 200 $\Omega$ cm. (**b**) The maximal slope of the depolarization upstroke (${\max}_{t} \frac{\partial v_{\mathrm{m}}}{\partial t}$) with respect to IR. It’s behavior is similar to that of CV. (**c**) The upstroke duration with respect to IR. Upstroke duration increases with increasing IR. (**a**)–(**c**) The black lines identify the high intercellular coupling domain, and the blue lines identify the low intercellular coupling domain. Data points are shown at IR = 1, 5, 10, 25, 50, 100, and 150 $\Omega$ cm.

Electrical conduction behaves differently for high and low intercellular coupling, modeled by IR values above and below 25 $\Omega$ cm, respectively. In the high intercellular coupling domain, CV is very sensitive to IR value changes, whereas CV has a low sensitivity for IR in the low intercellular coupling domain (Fig. 2a). Conduction block first occurs at 200 $\Omega$ cm. For IR values above that threshold, a stimulus propagates to only a few cells. From the reported data on CV and our simulations, we infer that the IR in excitable myometrium ranges between 10 and 100 $\Omega$ cm.

As intercellular coupling increases, membrane depolarization becomes faster. The maximal upstroke slope increases with increasing intercellular coupling (Fig. 2b), while the upstroke duration decreases with increasing intercellular coupling (Fig. 2c). As with conduction velocity, the sensitivity of the upstroke slope, and its duration, to intercellular coupling is high in the high intercellular coupling domain and low in the low intercellular coupling domain.

### Force development dependence on intercellular coupling

**Figure 3:**
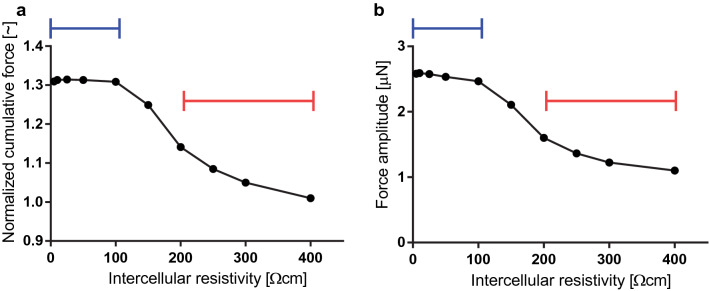
Force development with respect to intercellular resistivity (IR). (**a**) The normalized cumulative force developed by the myofibre is stable at low IR values, but it drops sharply as the IR approaches 200 $\Omega$ cm, where conduction block occurs. Then it remains at unity, reflecting that no force is generated besides passive tension. The normalized cumulative force value is defined as the integral of the force over 14 s after the initial stimulus, normalized by the passive force (i.e., without stimulation) developed by the myofibre over the same time period. (**b**) The amplitude of the force generated follows a similar pattern as the normalized force. (**a**,**b**) The blue lines mark the buffered domain, and the red lines mark the conduction block domain. Data points are shown are at IR = 1, 5, 10, 25, 50, 100, 150, 200, 250, 300, and 400 $\Omega$ cm.

As long as conduction is unblocked, the tensile force developed by the myofibre model is insensitive to IR. At every time step, our model calculates the tensile force developed by the myofibre in response to an initial stimulus. The forces developed under different IR values are compared by their amplitudes and normalized cumulative force values, defined as the integral of the force over 14 s after the initial stimulus, normalized by the passive force (i.e., without stimulation) developed by the myofibre over the same time period. This time period is the shortest time for which all the simulations return to their initial state after stimulation. We calculated the normalized cumulative force (Fig. 3a), as well as the amplitude of the force developed (Fig. 3b), for different IR values. The force developed has a weak dependence on IR for low IR values, but drops sharply around $200 \Omega \hbox {cm}$, where conduction block occurs. For higher IR values, the normalized area under the curve approaches unity; there is no force developed besides passive tension.

The robustness of the force developed against IR arises from ionic channel dynamics. The AP adapts to changing conduction velocities, buffering the calcium influx and consequently conferring insensitivity to the force developed from IR. The cellular dynamics of a cell in the middle of the myofibre explain this phenomenon. We consider the AP, the calcium influx, and the force-producing mechanism for the 30th cell in a 70-cell-long myofibre. The myofibre is stimulated as mentioned in the model overview, and the IR is varied between 10 and 400 $\Omega$ cm. For IR below 200 $\Omega$ cm, the entire myofibre is excited, at 300 $\Omega$ cm the cell selected is in the area just before conduction block occurs, and at 400 $\Omega$ cm the cell is past the conduction block.

**Figure 4:**
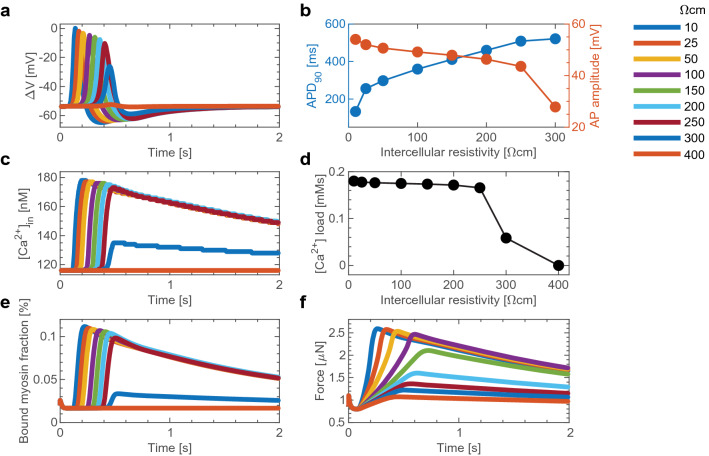
Mechanism for force robustness against intercellular resistivity (IR). (**a**) Simulated action potential (AP) traces for various IR values (see figure’s legend) at the 30th cell in a 70 cell long myofibre. (**b**) The action potential duration at 90% repolarization ($\mathrm{APD_{90}}$) and the AP amplitude of the traces in (**a**). (**c**) The intracellular calcium levels for the same conditions as in (**a**). (**d**) The calcium load over 14 s of simulation, calculated as the area under the curve of the traces in (**c**). (**e**) The fraction of myosin bound to actin over time. (**f**) The tensile force developed by the myofibre over time.

As the IR increases, conduction velocity decreases, and therefore the AP at the 30th cell is progressively delayed. The waveform amplitude is largely decreased just before conduction block, and the cell fails to activate for 400 $\Omega$ cm (Fig. 4a). The waveforms obtained for normal propagation are compared by the action potential duration at 90% repolarization ($\mathrm{APD_{90}}$), defined as the time required for the cell to restore to 90% its membrane potential, and through the AP amplitude. As the IR increases, the $\mathrm{APD_{90}}$ increases while the amplitude decreases (Fig. 4b). These two trends work in tandem to buffer the force driving calcium inward.

The intracellular calcium trace is characterized by a sharp upstroke followed by a slow inactivation (Fig. 4c). The increased IR delays the onset of the upstroke, in agreement with the AP delay. The intracellular calcium trace pattern responds to the AP dynamics: as the IR increases, the calcium influx current’s amplitude decreases, while it’s duration prolongs (Supplementary Fig. S2). These balancing forces keep the decay slope unchanged for varying IR values. The result is a buffered calcium load, defined as the area under the curve of the calcium transient, as long as conduction is unblocked (Fig. 4d).

By controlling the cross-bridge cycling dynamics, the intracellular calcium concentration regulates the force developed. Detailed descriptions of this mechanism can be found in the review by Aguilar et al.^29^. Briefly, myosins cycle through multiple states, and as they cycle, they attach and detach from actin filaments, producing a contractile force on the cell. The fraction of myosin bound to actin determines the force developed (Fig. 4e). In smooth muscles, the cycling of myosin through its multiple states is regulated by the myosin light chain kinase (MLCK), and MLCK’s activity, in turn, is regulated by the intracellular calcium concentration. This system was modeled by Yang et al.^19^ and included in our model (as detailed in the “Methods” section). The fraction of myosin in the force-producing states over time follows the intracellular calcium dynamics.

The force developed by the myofibre is calculated through our system of coupled equations (see “Model overview” and “Methods”). The bound myosin fractions of the cells in the myofibre determine the force developed by the myofibre (Fig. 4f). With increasing IR values, the force upstroke losses its sharpness and the peak force is attained at a later time. The buffered cross-bridge cycling dynamics maintain the force amplitude and normalized cumulative force for IR values below 150 $\Omega$ cm. This buffer effect is lost for IR values above 150 $\Omega$ cm, which is expected since conduction block occurs at 200 $\Omega$ cm (Fig. 3).

### Electrical conduction dependence on cellular excitability

Uterine smooth muscle cell excitability is mainly supported by calcium currents. The AP initial depolarization is driven by a long-lasting calcium current (I_CaL_), with a secondary transient calcium current (I_CaT_) and sodium current (I_Na_)^12,30,31^. This configuration differs from the well-studied cardiac and neural systems, where a single rapid sodium current is responsible for depolarizing the cell. Calcium channels in USMC require a sustained stimulus to activate and initiate the AP. The depolarizing stimulus for a given cell is provided by its adjacent depolarized cell through the GJ connecting both cells.

USMC excitability is highly regulated, but the effect of cellular excitability modulation on electrical conduction is incompletely understood. Ion-channels responsible for USMC excitability are upregulated as parturition approaches. Common uterotonic drugs, such as oxytocin, enhance cellular excitability to induce slowly progressing labours. On the other hand, tocolytic drugs are ion-channel blockers that aim to inhibit cellular excitability^32^.

**Figure 5:**
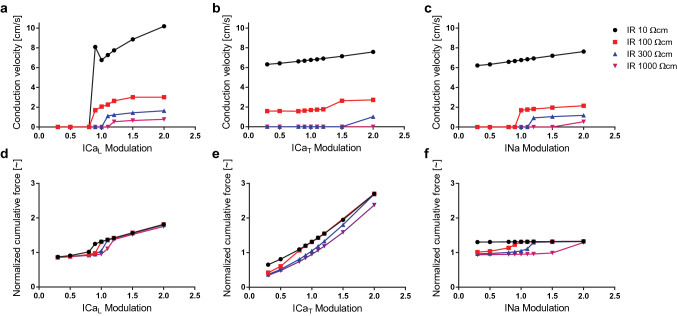
Electrical conduction and force development dependency on cellular excitability. (**a**–**c**) Conduction velocity (CV) as a function of I_CaL_, I_CaT_, and I_Na_ conductivity modulation, respectively. A zero CV indicates conduction block. Ionic channel current modulation is implemented as a multiplicative factor of the ionic channel conductivity: a modulation of 1.0 represents normal conductivity. The simulations were run under varying coupling levels, IR = 10, 100, 300, and 1000 $\Omega$ cm. (**d**–**f**) Normalized cumulative force developed by the myofibre under varying intercellular coupling levels and conductivity modulation of I_CaL_, I_CaT_, and I_Na_, respectively. Data points are shown at modulation = 0.3, 0.5, 0.8, 0.9, 1, 1.1, 1.2, 1.5, and 2.

To study the dependency of electrical conduction and force development on intercellular coupling and cellular excitability, we simulated the AP propagation through a myofibre with varying IR values, while modulating the cell’s conductivity for either I_CaL_, I_CaT_, or I_Na_ (Fig. 5).

Conduction block caused by low intercellular coupling is overcome with increasing cellular excitability. Increasing the conductance of the ion channels responsible for the I_CaL_ (Fig. 5a), I_CaT_ (Fig. 5b), or I_Na_ (Fig. 5c) currents restores conduction for IR values above the threshold for conduction block, and the cellular excitability upregulation required to restore conduction increases with decreasing intercellular coupling. Conduction block is most sensitive to I_CaL_ currents: small I_CaL_ downregulations block conduction even in well coupled myofibres, while small upregulations overcome blockage in poorly coupled myofibres.

Although conduction blockage is restored with increased cellular excitability, the conduction velocity is not. With increased cellular excitability, the conduction velocity of poorly coupled myofibres is considerably smaller than that of well coupled myofibres with normal cellular excitability. For example, increasing the conductivity of I_CaL_ in myofibres with IR above 100 $\Omega$ cm leads to only marginal increases in the conduction velocity (Fig. 5a). Within the physiological range of our model, CV is less sensitive to cellular excitability changes than to intercellular coupling changes.

### Force development dependence on cellular excitability

The effect of cellular excitability modulation on force development depends on the type of electrical current. In this study we explored the dependency on the main depolarizing currents, i.e., I_CaL_, I_CaT_, and I_Na_. As the conductivity for I_CaL_ increases, but does not overcome the conduction block, the force developed remains at the passive tension level. Then, as the conduction block is overcome, the force developed increases rapidly. After that, the force continues to increase gradually with increasing I_CaL_ conductivity (Fig. 5d).

The force developed increases for the entire range of I_CaT_ conductivity modulation (Fig. 5e). As conduction block is overcome, the plot of the force developed with respect to I_CaT_ modulation switches from the lower to the upper curve.

The force developed has a binary behavior with respect to I_Na_ conductivity modulation (Fig. 5f). The force remains at the passive tension level while conduction is blocked, then takes a higher value after the conduction block is overcome. Further increasing the I_Na_ conductivity, however, does not lead to increasing tensile forces.

For all current types, the force developed before and after overcoming the conduction block is independent of the intercellular coupling. Lower intercellular coupling implies that a higher cellular excitability increase is required to overcome the conduction block. However, once the conduction block is overcome, the force developed depends on I_CaL_ and I_CaT_ conductivity, and not on intercellular coupling.

## Discussion

We developed a uterine myofibre model and used it to explore electrical conduction and force development. This model integrates validated models of membrane currents, calcium dynamics, and force development in a novel mathematical framework. A coupled system of differential equations governs the propagation of the electromechanical wave along a series of cells modelling a myofibre. To study the regulation of electrical conduction and force development in the myometrium, we simulated the propagation of a stimulus under varying conditions of intercellular coupling and cellular excitability.

Our model simulation showed that intercellular coupling is the main regulator of CV. Very low intercellular coupling induces conduction block, and for higher intercellular coupling we identified two domains. In the low intercellular coupling domain, we observed a slow CV that is insensitive to intercellular coupling changes. On the other hand, in the high intercellular coupling domain, we observed a high CV that is also very sensitive to intercellular coupling. Although cellular excitability changes, simulated by modulation of the ion channels’ conductance, can recover conduction block caused by low intercellular coupling, the effect of those changes on CV is marginal. Our simulations also showed that the force developed is buffered for varying intercellular coupling values, provided the stimulus propagates along the entire myofibre. The simulated traces for the voltage, calcium, and myosin state explain this compensatory mechanism. Lastly, we explored the effect of cellular excitability on force development and found that upregulating the conductivity of I_Na_ and I_CaL_ has a minimal effect on the force developed, once conduction is established. Upregulating I_CaT_, on the other hand, has a profound effect on the force developed.

Our results contribute to a better understanding of uterine electrophysiology. To the best of our knowledge, this is the first computational study to explore and characterize the propagation of the electromechanical wave in the myometrium. We studied the effects of intercellular coupling and cellular excitability on electrical propagation and force development. Intercellular coupling and cellular excitability are remodeled at the end of pregnancy, in preparation for parturition. Moreover, all the available tocolytic and uterotonic drugs target these mechanisms. Therefore, the exploration of these mechanisms is fundamental to understanding the remodelling processes of the myometrium and to developing more efficient and safer drugs to modulate them.

All models, including this one, have limitations. Although multiple AP morphologies have been reported in the myometrium^33^ , we considered only a single AP morphology. Our simulations are based on the spiked AP elicited by a brief depolarization of a USMC. Tong et al.^12^ showed that their USMC model, which was used in this study, can reproduce multiple AP morphologies and the effects of AP trains. Our study explores the intrinsic properties of the myofibre but not the dependency on the AP morphology. Additionally, multiple ionic current models of USMC are available. We chose to use the model of Tong et al. based on their experimental validation and a preliminary stability analysis. Our framework, however, is modular, and the USMC ionic current model can be replaced with newer models.

An important limitation of our model is that it has been developed from available smooth muscle models, rather than from dedicated experimental observations. Firstly, the models integrated in our study were developed from experimental observations recorded by several groups using different experimental systems and species. Secondly, the models used to describe myosin cycling^20^ and the subsequent cellular contraction^19^, were developed for vascular smooth muscles. In the future, as experimental and theoretical uterine electrophysiology advances, uterine excitation-contraction models will preferably be developed based on consistent experimental observations and theoretical frameworks.

Future extensions of our study include investigating other regulatory mechanisms of conduction and force development in the myometrium. In particular, we note that the myometrium expresses stretch activated potassium channels. These channels open in response to stretch and inhibit membrane excitability. Multiple groups have shown that these ion channels are expressed during pregnancy and downregulated towards term. This suggests that these ion channels play a role in maintaining the quiescent electrical state of the uterus during pregnancy^11,34,35^.

Moreover, other mechanosensitive ion channels have been found in the myometrium, e.g., swell-activated chloride channels, non-specific cation channels, and calcium channels^11^. Our model implements the ion channel model developed by Tong et al.^12^ but does not incorporate mechanosensitive ion channels. In the future, this ion channel model could be replaced for another that includes mechanosensitive ion channels to study in the electromechanical feedback in further detail.

Additionally, we are interested in the effects of myosin light chain kinase regulation. This kinase, regulated by oxytocin and progesterone, is thought to be a key regulator of myometrial excitability and contraction^10,36^. Lastly, while uterine APs usually appear in bursts and our model can simulate the bursting type AP^12,13^, as shown in Supplementary Fig. S3, we only used single spiked APs for our simulations. Using single APs simplifies the analysis of the mechanisms underlying the excitability and contractility of the myofibre. However, additional complex observations can be made from analyzing the propagation of the bursting type AP. Further study of these mechanisms may help prevent preterm birth and assist in labor induction.

## Methods

### USMC model

We model a myofibre as a longitudinal concatenation of USMC ionic models. Each cell is represented by the detailed ionic current model developed by Tong et al.^12^. The complete formulation and implementation of this model can be found in the original publication. Briefly, the ionic currents, the transmembrane voltage, and the intracellular calcium concentration are related through a system of over 100 differential equations. This model contains 15 different ionic currents, most of which are modeled through the Hodgkin Huxley formalism, i.e.,

1 $$\begin{aligned} I= & {} \bar{g}\prod _{i=1}^{n} y_i(v-E_{\mathrm{rev}}) , \end{aligned}$$

2 $$\begin{aligned} E_{\mathrm{rev}}= & {} \frac{RT}{F} \mathrm{ln} \frac{[X]_{\mathrm{o}}}{[X]_{\mathrm{i}}} , \end{aligned}$$

3 $$\begin{aligned} \frac{\mathrm{d}y_i}{\mathrm{d}t}= & {} \frac{y_{{\infty }_i} - y_i}{\tau _{y_i}} , \end{aligned}$$

where *I* is the transmembrane ion current, $\bar{g}$ is the maximal conductance for the given current, $y_i$ are the gating variables, *v* is the membrane potential, $E_{\mathrm{rev}}$ is the Nernst potential for the ion X, *R* is the gas constant, *T* is the absolute temperature, *F* is the faraday constant, $[X]_{\mathrm{o}}$ and $[X]_{\mathrm{i}}$ are the extracellular and intracellular ion concentrations, and $y_{{\infty }_i}$ and $\tau _{y_i}$ are the steady state and time constants for channel *i* and depend on the transmembrane voltage and intracellular calcium concentration.

### Excitation model

A depolarizing current is injected into the first cell in a series and triggers an AP. The depolarization of USMCs results mostly from inward calcium currents and promotes the depolarization of the following cell through their electrical coupling. The resulting depolarization wave is governed by the cable equation. Assuming an homogenous extracellular medium, the cable equation is given by^37^

4 $$\begin{aligned} \partial _x(\sigma \partial _xv(x,t)) = \chi ( C_{\mathrm{m}}\partial _t v(x,t) + I_{\mathrm{ion}}(x,t) + I_{\mathrm{stimulus}}(x,t) ) , \end{aligned}$$

where *V*(*x*, *t*) is the transmembrane voltage along the cable; $I_{\mathrm{ion}}(x,t)$ is the ionic current based on the model of Tong et al.; $I_{\mathrm{stimulus}}(x,t)$ is the stimulatory current, which in our case is spatially constrained to the first cell; $C_{\mathrm{m}}$ is the specific membrane capacitance; $\sigma$ is the intercellular conductivity; and $\chi$ is the cell’s surface to volume ratio.

We assume that the cells’ volume and surface are deformable but incompressible. In other words, while the cells’ shape can change, the total volume and surface area remain constant. Therefore, in our model the surface to volume ratio $(\chi )$ remains constant.

The intercellular coupling is the product of the intercellular cross sectional area, $A_{\mathrm{ics}}$, that is the cross sectional area at the intercellular junction, and the intercellular conductivity, $\sigma$. The intercellular coupling is inversely proportional to IR, denoted by $\rho$.

5 $$\begin{aligned} \mathrm{Interellular\ coupling} :=A_{\mathrm{ics}}\sigma =\frac{A_{\mathrm{ics}}}{\rho } . \end{aligned}$$

In our model, we assume that the intercellular cross sectional area, $A_{ics}$, remains constant throughout the simulation. This assumption is supported by the fact that the intercellular junctions are rich in anchoring protein complexes that hold the membrane’s surface area at the intercellular junction. We do not assume, however, that the cellular cross sectional area remains constant throughout the cell. On the contrary, the cellular cross sectional area can change, under the constraints that the total volume, total surface area, and cross sectional area at the intercellular junction remain constant.

### Cellular contraction model

$[Ca^{2+}]_{\mathrm{in}}$ regulates the fraction of myosin in each of its four possible states, as described by Hai and Murphy^20^. The four states are free unphosphorylated (M), phosphorylated (Mp), phosphorylated cross bridges (AMp) and latch (AM), where AMp and AM are the force-generating states. Here, *M*, *Mp*, *AMp*, and *AM* represent the fraction of myosin in each state, respectively. The transition between the states is governed by the following transition system:

6 $$\begin{aligned} \begin{pmatrix} \frac{\mathrm{d}M}{\mathrm{d}t}\\ \frac{\mathrm{d}Mp}{\mathrm{d}t}\\ \frac{\mathrm{d}AMp}{\mathrm{d}t}\\ \frac{\mathrm{d}AM}{\mathrm{d}t}\\ \end{pmatrix} = \begin{pmatrix} -K_1 &{} K_2 &{} 0 &{} K_7\\ K_1 &{} -K_2-K_3 &{} K_4 &{} 0\\ 0 &{} K_3 &{} -K_4 - K_5 &{} K_6\\ 0 &{} 0 &{} K_5 &{} -K_6-K_7\\ \end{pmatrix} \begin{pmatrix} M\\ Mp\\ AMp\\ AM\\ \end{pmatrix}, \end{aligned}$$

where $K_1$ and $K_6$ are regulated by $[Ca^{2+}]_{\mathrm{in}}$:

7 $$\begin{aligned} K_1 = K_6 = \frac{[Ca^{2+}]_{\mathrm{in}}^{nm}}{[Ca^{2+}]_{\mathrm{in}}^{nm} + C_aMLCK^{nm}} . \end{aligned}$$

Here, *nm* is the Hill coefficient; *CaMLCK* is the half-saturation concentration of MLCK; and $K_2$, $K_3$, $K_4$, $K_5$, and $K_7$ are myosin state transition constants.

We modeled the contractile mechanics of a single cell using the model developed by Yang et al.^19^. In this model, the force is developed by a passive element connected in parallel to three serially connected force-generating elements. The force-generating elements are the cross bridge elasticity, the active force-generating element, and the series viscoelastic element. A listing of the mechanical parameters appears in Table 1.

**Table 1:** Definitions of the mechanical parameters used in the derivation of the myofibre contraction model. The parameters were adapted from the model developed by Testrow et al.^13^.

Symbol	Parameter description
$K_{\mathrm{p}}$	Parallel element stiffness constant
$\alpha_{\mathrm{p}}$	Length modulation for passive element
$l_{\mathrm{c}}$	Cell’s length
$l_{\mathrm{0}}$	Length of cell at zero passive force
$l_{\mathrm{a}}$	Length of active component of cell
$l_{\mathrm{s}}$	Length of spring component of cell
$l_{\mathrm{x}}$	Length of cross bridge component of cell
$l_{\mathrm{opt}}$	Optimal length of active contractile component
$l_{\mathrm{s0}}$	Length of series viscoelastic component at zero force
$K_{\mathrm{x1}}$	Phosphorylated cross-bridge stiffness constant
$K_{\mathrm{x2}}$	Latch bridge stiffness constant
$\beta$	Length modulation constant for active and cross-bridge elements
$f_{\mathrm{AMp}}$	Friction constant for phosphorylated cross-bridge
$f_{\mathrm{AM}}$	Friction constant for latch bridges
$\nu_{\mathrm{x}}$	Cross-bridge cycling velocity
$\mu_{\mathrm{s}}$	Viscosity coefficient of series element
$k_{\mathrm{s}}$	Series element stiffness constant
$\alpha _{\mathrm{s}}$	Length modulation for series viscoelastic element
$l_{\mathrm{c0}}$	Initial cell’s length

The passive force (p) is given by

8 $$\begin{aligned} F_{\mathrm{p}} = K_{\mathrm{p}}\left( e^{\alpha _{\mathrm{p}} \frac{l_{\mathrm{c}} - l_{\mathrm{0}}}{l_{\mathrm{0}}}} -1\right) . \end{aligned}$$

The force generated by the cross bridge elasticity (x) is given by

9 $$\begin{aligned} F_{\mathrm{x}} = \left( k_{\mathrm{x1}}AMp + k_{\mathrm{x2}}AM\right) l_{\mathrm{x}}e^{-\beta \left( \frac{l_{\mathrm{a}} - l_{\mathrm{opt}}}{l_{\mathrm{opt}}}\right) ^2}. \end{aligned}$$

The active force (a) generated is given by

10 $$\begin{aligned} F_{\mathrm{a}} =\left[ f_{\mathrm{AMp}}AMp\left( \nu _{\mathrm{x}} + \frac{\mathrm{d}l_{\mathrm{a}}}{\mathrm{d}t}\right) + f_{\mathrm{AM}}AM\frac{\mathrm{d}l_{\mathrm{a}}}{\mathrm{d}t}\right] e^{-\beta \left( \frac{l_{\mathrm{a}} - l_{\mathrm{opt}}}{l_{\mathrm{opt}}}\right) ^2}. \end{aligned}$$

The series viscoelastic force (s) is given by

11 $$\begin{aligned} F_{\mathrm{s}} = \mu _{\mathrm{s}} \frac{\mathrm{d}l_{\mathrm{s}}}{\mathrm{d}t} + k_{\mathrm{s}}\left( e^{\alpha _{\mathrm{s}} \frac{l_{\mathrm{s}} - l_{\mathrm{s0}}}{l_{\mathrm{s0}}}} -1\right) . \end{aligned}$$

Since the length of the cell (*l*c) is spanned by the serially connected elements, we can write

12 $$\begin{aligned} l_{\mathrm{c}} = l_{\mathrm{a}} + l_{\mathrm{x}} + l_{\mathrm{s}} . \end{aligned}$$

Using Newton’s third law we get

13 $$\begin{aligned} F_{\mathrm{s}} = F_{\mathrm{a}} = F_{\mathrm{x}} . \end{aligned}$$

The tensile force generated by the cell is given by

14 $$\begin{aligned} F_{\mathrm{t}} = F_{\mathrm{p}} + F_{\mathrm{a}} . \end{aligned}$$

### Myofibre contraction model

For a travelling AP in a myofibre of $i=1\ldots n$ cells, the myosin cycling is unique to each cell and is dependent on its electrical activation. Our problem, then, is as follows: given $AM^i(t)$ and $AMp^i(t)$, we are interested in finding the lengths of each cell in the myofibre and the tensile force developed by the entire myofibre. To solve this problem, we need to find the lengths of the cellular compartments of each cell at every time step. For a myofibre of *n* cells, we get 3*n* unknowns: $l_{\mathrm{j}}^i(t)_{\mathrm{j}=\mathrm{a,s,x}}^{i=1\ldots n}$ .

Combining Eqs. (9), (10), and (13) we get

15 $$\begin{aligned} (k_{\mathrm{x1}}AMp^{(i)} + k_{\mathrm{x2}}AM^{(i)})l_{\mathrm{x}}^{(i)}(t) = f_{\mathrm{AMp}}AMp^{(i)} \left( \nu _{\mathrm{x}} + \frac{\mathrm{d}l_{\mathrm{a}}^{(i)}}{\mathrm{d}t} \right) + f_{\mathrm{AM}}AM^{(i)}\frac{\mathrm{d}l_{\mathrm{a}}^{(i)}}{\mathrm{d}t} \quad \forall \,i = 1\ldots n. \end{aligned}$$

Combining Eqs. (9), (11), and (13), we then get

16 $$\begin{aligned} (k_{\mathrm{x1}}AMp^{(i)} + k_{\mathrm{x2}}AM^{(i)})l_{\mathrm{x}}^{(i)}e^{-\beta \left( \frac{l_{\mathrm{a}}^{(i)} - l_{\mathrm{opt}}}{l_{\mathrm{opt}}}\right) ^2} = \mu _{\mathrm{s}} \frac{\mathrm{d}l_{\mathrm{s}}^{(i)}}{\mathrm{d}t} + k_{\mathrm{s}}\left( e^{\alpha _{\mathrm{s}} \frac{l_{\mathrm{s}}^{(i)} - l_{\mathrm{s0}}}{l_{\mathrm{s0}}}} -1\right) \quad \forall \,i = 1\ldots n . \end{aligned}$$

From Newton’s third law we can write that

17 $$\begin{aligned} F_{\mathrm{t}}^{(i)} = F_{\mathrm{t}}^{(i+1)} \qquad \quad \forall \,i = 1\ldots n-1. \end{aligned}$$

Combining Eqs. (14) and (17), we get

18 $$\begin{aligned} &(k_{\mathrm{x1}}AMp^{(i)} + k_{\mathrm{x2}}AM^{(i)})l_{\mathrm{x}}^{(i)}e^{-\beta \left( \frac{l_{\mathrm{a}}^{(i)} - l_{\mathrm{opt}}}{l_{\mathrm{opt}}}\right) ^2} + K_{\mathrm{p}}\left( e^{\alpha _{\mathrm{p}} \frac{l_{\mathrm{c}}^{(i)} - l_{\mathrm{0}}}{l_{\mathrm{0}}}} -1\right) \\&\quad = (k_{\mathrm{x1}}AMp^{(i+1)} + k_{\mathrm{x2}}AM^{(i+1)})l_{\mathrm{x}}^{(i+1)}e^{-\beta \left( \frac{l_{\mathrm{a}}^{(i+1)} - l_{\mathrm{opt}}}{l_{\mathrm{opt}}}\right) ^2} + K_{\mathrm{p}} \left( e^{\alpha _{\mathrm{p}} \frac{l_{\mathrm{c}}^{(i+1)} - l_{\mathrm{0}}}{l_{\mathrm{0}}}} -1\right) \quad \forall \,i = 1\ldots n-1. \end{aligned}$$

The last equation needed to solve the system comes from the isometric condition. The myofibre’s total length, but not an individual cell’s length, is held constant. This condition is justified because, rather than being driven by uterine volume changes, intrauterine pressure buildup is driven by increases in wall tension, as described by Laplace’s law^38^:

19 $$\begin{aligned} \sum _{i=1}^{n} \,l_{\mathrm{a}}^{(i)} + l_{\mathrm{x}}^{(i)} + l_{\mathrm{s}}^{(i)} = n\cdot l_{\mathrm{c0}}. \end{aligned}$$

Since the length of every cell is given by

20 $$\begin{aligned} l_{\mathrm{c}} ^{(i)} = l_{\mathrm{a}}^{(i)} + l_{\mathrm{x}}^{(i)} + l_{\mathrm{s}}^{(i)}, \end{aligned}$$

Eq. (19) can be rewritten as

21 $$\begin{aligned} \sum _{i=1}^{n} \,l_{\mathrm{c}}^{(i)} = n\cdot l_{\mathrm{c0}} . \end{aligned}$$

We then have a nonlinear system of 3n unknowns and equations that needs to be solved at every time step. Solving this system, together with the cable equation, which includes the ionic current model of each cell in the myofibre, gives us the transmembrane voltage, the intracellular calcium concentrations, the length of each cell, and the force developed by each cell.

Since all the model’s variables are coupled, the entire system described by the equations in the “Methods” section needs to be solved at every time step to calculate the model outputs. For example, to calculate the length of every cell ($l_{\mathrm{c}}^{(i)}$) from Eq. (20), the internal cells’ lengths ($l_{\mathrm{a}}^{(i)}$, $l_{\mathrm{x}}^{(i)}$, and $l_{\mathrm{s}}^{(i)}$) need to be calculated using Eqs. (15–19), which in turn require calculating the myosin phosphorylation states (Eqs. 6 and 7), and the cable Eq. (4), which requires solving the cells’ ionic currents (Eqs. 1–3). Due to the complexity of this system, it is solved using the GNU scientific library in C++^39^.

The excitation and contraction waves are coupled. While the electrical activity produces mechanical deformations and tensile force, the mechanical deformations feed back into the electrical activity through the geometrical parameters of the cable equation. This feedback is obtained through the left hand side of the cable equation, shown in Eq. (4).

To solve the cable Eq. (4) at every time step, we discretize the myofibre and consider each cell as an infinitesimal element. Using discrete operators, the left hand side of Eq. (4) becomes:

$$\begin{aligned} \sigma \frac{v^{(i+1)}(t) -2v^{(i)}(t) + v^{(i-1)}(t) }{ \left( l_{\mathrm{c}}^{(i)}(t)\right) ^2} \end{aligned}$$

where the superscript (*i*) denotes the *i*th cell in the myofibre. This expression closes the feedback loop since the length of the *i*th cell in the myofibre is used to calculate the electrical propagation across that cell and depends on the mechanical state of that cell.

## Supplementary information

Supplementary Figures.

Supplementary Information.

## Data Availability

The software used for this simulations is publicly available at Github: https://github.com/uri-goldsztejn/uterine_myofibre_model.git
