# Supplementary material for: A myofibre model for the study of uterine excitation-contraction dynamics
Source: Sci Rep. 2020 Oct 1;10:16221. doi: 10.1038/s41598-020-72562-x (PMC7530703; doi:10.1038/s41598-020-72562-x)
Supplement: Supplementary file 1 — Supplementary Figures. [file 41598_2020_72562_MOESM1_ESM.pdf]

A myofibre model for the study of uterine excitation-contraction dynamics

Uri Goldsztejn[1] **and Arye Nehorai**[2,*]

[1]Washington University in St. Louis, Department of Biomedical Engineering, St. Louis, 63130, USA
[2]Washington University in St. Louis, Department of Electrical and Systems Engineering, St. Louis, 63130, USA
[*]nehorai@wustl.edu

Supplementary information

Supplementary figure S1

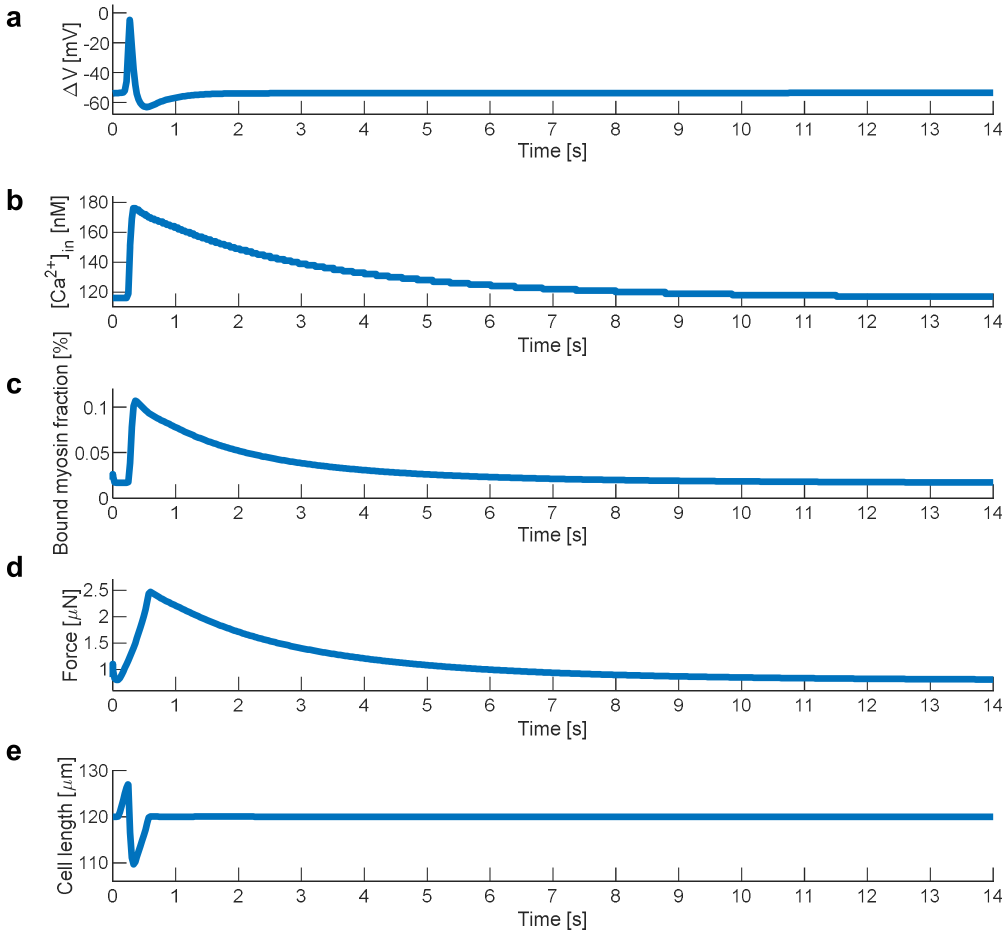

Full length simulation. A 70 cell long myofibre was stimulated from one end with a 30 ms long depolarization stimulus of -5 pA/pF. The intercellular resistivity of the myofibre is 100 Ωcm. The traces shown correspond to the internal parameters of the 30th cell. Our model returns to its initial state in less than 14 s after stimulation. **(a)** The transmembrane voltage shows a spiked action potential (AP). **(b)** The intracellular calcium concentration rises sharply after the onset of the AP but has a slow recovery. **(c)** The fraction of myosin bound to actin determines the contractile force developed and is regulated by the intracellular calcium dynamics. **(d)** The contractile force developed peaks after the peak of the fraction of myosin bound to actin due to the viscous components of the contractile cellular mechanism. **(e)** The cell expands as its upstream neighbours contract. Then, it contracts as its myosin binds to actin. After the AP travels across the myofibre, the cells return to their initial lengths but continue producing a higher tensile force.

Supplementary figure S2

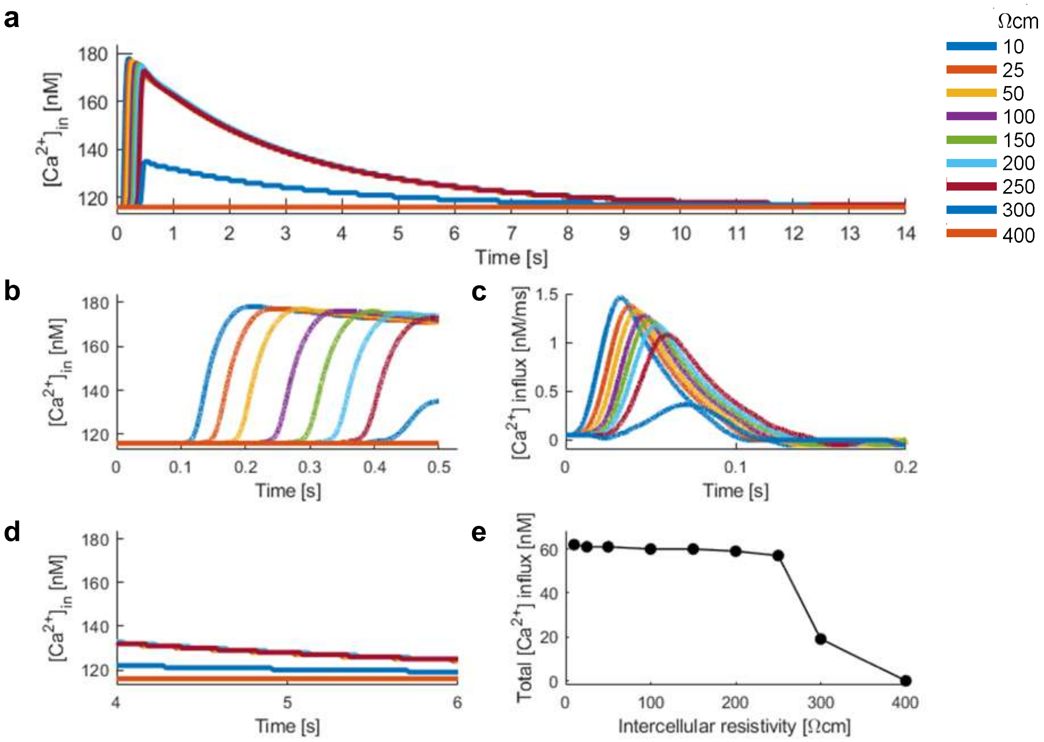

Calcium flux dynamics with respect to intercellular resistivity. The simulation results correspond to the 30th cell of a 70 cell long myofibre stimulated with a 30 ms long depolarization stimulus of -5 pA/pF. **(a)** The intracellular calcium transients obtained after depolarization. The calcium transient waveforms are mostly unchanged while there is no conduction block. When the intercellular resistivity is larger than 250 Ωcm, the waveform amplitude decreases considerably. **(b)** An enlarged view of the calcium transient's upstroke. As the intercellular resistivity increases, and before it produces conduction block, the upstroke is delayed in time in agreement with the reduced conduction velocity. **(c)** The calcium influx current, calculated as the time derivative of the traces in (**b**). For comparison, all the traces where shifted in time to start at the same timepoint. As the intercellular resistivity increases, the calcium influx current amplitude decreases and its duration increases. The trace obtained for intercellular resistivity of 400 Ωcm is not shown here for clarity. It's derivative is 0 over the entire doamin. **(d)** An enlarged view of the calcium transients during the decay phase. The decay slopes overlap as long as there is no conduction block. **(e)** Total calcium influx, calculated as the area under the curve of the traces in (**a**), minus the area obtained with the resting intracellular calcium concentration. The total calcium influx is mostly unchanged as long as there is no conduction block.

Supplementary figure S3

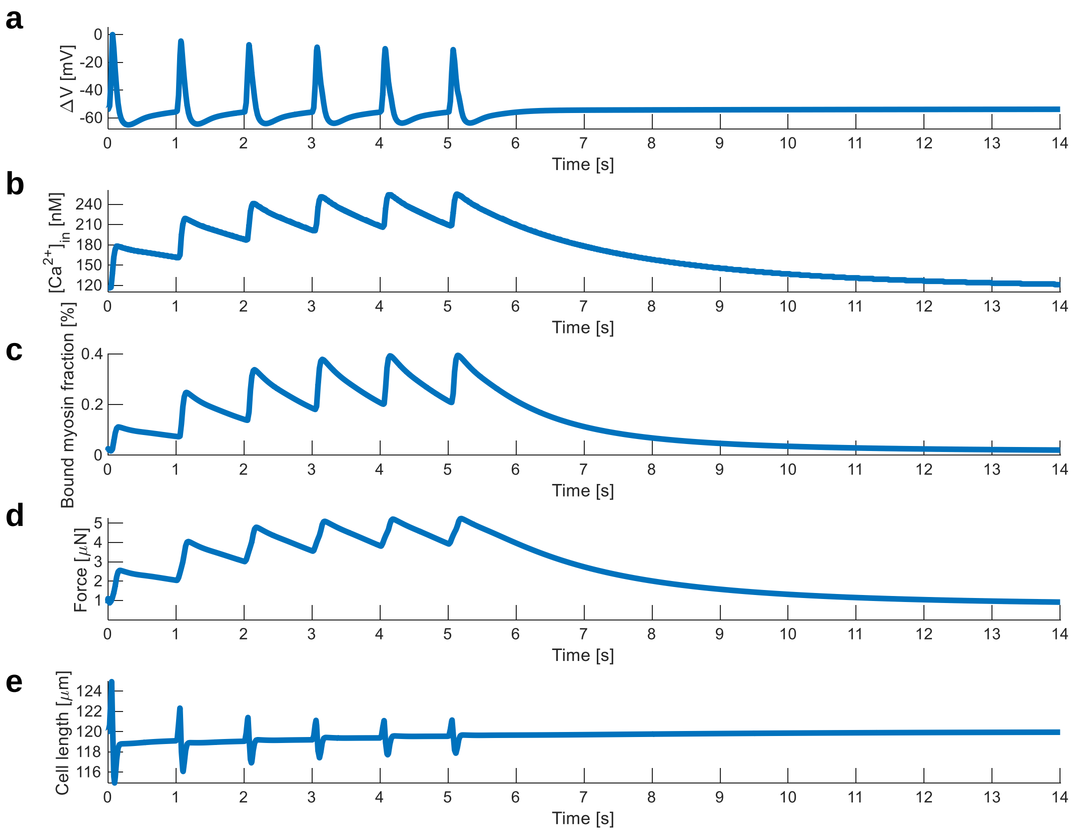

Burst stimulation. Bursts of APs appear in the contracting myometrium. To simulate the bursting type AP, we stimulated a 20 cell long myofibre from one end with 6 consecutive depolarizing stimuli. The stimuli were 30 ms long, injected a current of -5 pA/pF, and were activated at a rate of 1 Hz. We used a shorter myofibre than in the previous simulations, because consecutive APs are fired before the cells fully repolarize and therefore travel a shorter distance. The intercellular resistivity of the myofibre is 100 Ωcm. The traces shown correspond to the internal parameters of the 10th cell. Our model returns to its initial state about 14 s after stimulation. **(a)** The transmembrane voltage shows consecutive spiked APs. **(b)** The intracellular calcium concentration rises sharply after the onset of each AP and has a slow recovery. Therefore, intracellular calcium accumulates with consecutive APs. **(c)** The fraction of myosin bound to actin determines the contractile force developed and is regulated by the intracellular calcium dynamics. **(d)** The contractile force developed increases sharply with each AP and declines slowly. **(e)** With each AP, the cell expands as its upstream neighbours contract. Then, it contracts as its myosin binds to actin. Lastly, after the AP travels across the myofibre, the cells return to their initial lengths but continue producing a higher tensile force.
